# Neurovascular threats to brain health: implications for Abeta immunotherapy

**DOI:** 10.1002/alz70861_108866

**Published:** 2025-12-23

**Authors:** Costantino Iadecola

**Affiliations:** ^1^ Weill Cornell Medicine, New York, NY USA

## Abstract

**Background:**

Cerebrovascular health is a major determinant of brain health. The cerebral microvasculature not only meets the brain’s energy demands by delivering O2 and glucose but also maintains microenvironmental homeostasis. This is achieved through the blood–brain barrier, clearance of toxic byproducts of neural activity such as amyloid‐beta (Abeta) and tau, and the regulation of immune cell trafficking in and out of the brain. Given these essential roles, it is not surprising that vascular factors exert a profound influence on brain health. In the aging population, the most common cause of dementia is mixed dementia combining features of both vascular and Alzheimer disease (AD) pathology, both of which are exacerbated by arterial hypertension and ApoE4 carriage

**Method:**

N/A

**Result:**

This presentation will explore the cellular and molecular mechanisms through which AD pathology, ApoE4, and hypertension impair key regulators of cerebral microcirculation—specifically, neurovascular coupling, endothelial function, and interstitial fluid clearance. Special emphasis will be placed on neuroimmune interactions, particularly the emerging role of border‐associated macrophages (BAMs)—a distinct population of brain‐resident innate immune cells. Evidence will be presented that Aβ, ApoE4, and hypertension activate BAMs positioned adjacent to blood vessels, triggering vascular oxidative stress and inflammation, which, in turn, contribute to neurovascular dysfunction, impaired clearance of metabolic waste, and cognitive decline.

**Conclusion:**

Finally, the implications of these neurovascular and neuroimmune effects for the Amyloid Related Imaging Abnormality (ARIA) syndrome, a treatment limiting and potentially fatal complication of Abeta immunotherapy, will be examined.

